# Reliability and validity of the Japanese treatment self-regulation questionnaire for Japanese workers

**DOI:** 10.1186/s12889-022-14281-8

**Published:** 2022-10-11

**Authors:** Kayoko Ishii, Kumiko Morita, Hiroko Sumita

**Affiliations:** Tokyo Medical and Dental University (TMDU), 1-5-45 Yushima Bunkyo-ku, 113-8519 Tokyo, Japan

**Keywords:** Self-determination theory, Health behaviors, Motivation, Occupational health, Diet, Exercise

## Abstract

**Background:**

Motivational interventions are used as preventive measures in occupational health. However, existing studies primarily focus on motivation methods and not the stage of motivation—the process from extrinsic to intrinsic motivation. The treatment self-regulation questionnaire (TSRQ) can predict workers’ health at each motivational stage. Accordingly, this study examined the reliability and validity of the Japanese version of the TSRQ (Diet and Exercise) in occupational health settings.

**Methods:**

Responses of 912 workers were analyzed. In this study, the Cronbach’s alphas were 0.85 for Diet and 0.84 for Exercise after excluding items with low Item-Total correlations. Regarding convergent validity, there was a weak correlation between behavior modification stages and the TSRQ. Regarding structural validity, confirmatory factor analysis was performed assuming a four-factor structure.

**Results:**

The goodness-of-fit indices were: Comparative Fit Index (CFI) = 0.94, Tucker Lewis Index (TLI) = 0.92, and Root Mean Square Error of Approximation (RMSEA) = 0.07 for Diet and CFI = 0.92, TLI = 0.91, and RMSEA = 0.08 for Exercise.

**Conclusion:**

The Japanese version of the TSRQ has a certain degree of reliability and validity. It can measure motivation for Diet and health-related behaviors in occupational health settings. The findings of this study may serve as a basis for promoting primary and secondary prevention.

## Background

Non-communicable diseases (NCDs) or chronic diseases, including cardiovascular diseases (such as heart attacks and stroke), cancers, respiratory diseases (such as chronic obstructive pulmonary disease and asthma), and diabetes, are the primary causes of mortality in Japan [1]. The World Health Organization [2] defines NCDs as long-term illnesses caused by a combination of genetic, physiological, environmental, and behavioral factors. Lifestyle-related diseases refer to NCDs caused by lifestyle-related habits such as diet, exercise, sleep, smoking, and drinking, which influence the onset and progression of diseases. Lifestyle-related diseases account for 30% of total medical costs and 60% of all deaths in Japan; thus, preventive approaches are urgently needed [3]. In Japan, there has been a legal mandate since 2008 to provide specific health check-ups (SHC) and specific health guidance (SHG) for all those aged between 40 and 74 years [4, 5]. Such provisions are intended to prevent lifestyle-related diseases (lipid abnormalities, hypertension, diabetes) and improve lifestyles. SHG is given to those who are deemed in need of it based on the results of the SHC. Motivational support is a part of SHG and is another way to promote healthy behavior. However, previous studies [6] show that receiving SHG improves obesity by only 2%. Therefore, the Ministry of Health, Labour and Welfare (2022) [7] concluded that it is necessary to “visualize the process of behavior change” and to “assess the implementation of SHG (outcome assessment)” as future issues. Several companies in Japan also offer programs in addition to SHG to their workers to prevent lifestyle diseases. These include providing health guidelines to help workers maintain a healthy lifestyle from a young age. The current health guidelines provided in occupational health settings emphasize the importance of motivation in increasing workers’ awareness of their health condition, helping them understand the necessity of lifestyle improvements, and promoting the practice of health behaviors [8]. Although health guidelines have been incorporated into the “stages of change,” few studies have assessed the stage of motivation. The stages of change model, which is widely applied in Japan now, posits that individuals move through five stages of behavioral change: pre-contemplation, contemplation, preparation, action, and maintenance. This model can help reveal the stages of change, but not the stages of motivation that affect behavioral sustainability.

The treatment self-regulation questionnaire (TSRQ) resolves the problems of the SHG and supports the intentions of company health guidelines. It was developed by Ryan and Connell [9] to assess autonomous self-regulation and has since been used in a variety of settings, including healthcare. The reliability and validity of the scale have been verified in various countries, and it has been used to develop and implement interventions for behavior change [10, 11]. This questionnaire is based on self-determination theory (SDT), which comprises six sub-theories: cognitive evaluation, organismic integration, causality orientations, basic psychological needs, goal contents, and relationships motivation. The SDT hypothesizes that greater relative autonomy is associated with higher quality behavior and greater persistence [12]. The key point here is the improvement in autonomy. Particularly, organismic integration focuses on the value of an activity and captures the degree of relative autonomy, including the relationship between extrinsic and intrinsic motivation. In other words, this theory concerns the process of evolution from the extrinsic motivation stage to the intrinsic motivation stage [13]. The TSRQ evaluates this process [14]. The TSRQ is widely used for evaluating a patient’s degree of autonomy in undertaking changes in risky behaviors, introducing medical treatment, assessing its maintenance, and participating in a screening procedure for disease prevention [15]. Levesque et al. [10] validated the scale and confirmed its reliability and validity in the United States, Europe, and other countries. Silva et al. [16] conducted an SDT-based weight loss program for women aged 25–50 years and evaluated the program using the TSRQ. The results showed that the SDT intervention led to significant weight loss compared to other interventions [16]. These findings indicate the effectiveness of SDT as well as the usefulness of the TSRQ.

In Japan, too, the TSRQ can be used to determine the stage of motivation, which in turn can make it possible to evaluate interventions that aim to bring about health behavioral changes. By combining the stages of actual behavior and motivation, it is possible to provide tailored health guidance. The important thing in motivating behavioral change for health is to judging one’s own behavior andmatching and integrating one’s values and lifestyle patterns is crucial to motivating behavioral change for health [17]. Therefore, surrounding medical personnel have the role of aiding this change and providing support when encountering barriers. However, the current uniform set of standards and practices of SHG might not be sufficient to promote and sustain behavioral change for health[18]. Individuals can integrate healthy behavior into their lifestyle by engaging in tailor-made engagement according to the motivation stage, in addition to the uniform motivational interviewing currently practiced in Japan. In Japan, there is a large body of research on the use of SDT in the field of education. However, in healthcare, there has only been one study, which targeted patients on dialysis [19]. The TSRQ assesses various health behavior domains, including Diet, Exercise, Smoking, and Responsible Alcohol Consumption; each of these can be used independently. In this study, we used the Diet and Exercise questionnaires. Smoking and Responsible Alcohol Consumption were excluded as both cigarette smoking and alcohol consumption are expensive habits. Moreover, the annual smoking rate in Japan has decreased to 16%, and the prevalence of alcohol consumption, which increases the risk of developing lifestyle-related diseases, has been reported to be 14.9% for men and 9.1% for women, indicating a declining trend [20].

## Aim

In this study, we sought to assess the extent to which an individual is motivated to engage in diet and exercise-related behaviors and included subscales such as autonomous motivation, introjected regulation, external regulation, and amotivation [12]. Thus, the purpose of this study was to investigate the reliability and validity of the Japanese version of the TSRQ. We conducted the validation process based on the COSMIN checklist [21]. We hypothesized that the TSRQ has internal consistency, convergent validity, and structural validity and that the scores on the Japanese version of the TSRQ are correlated with the stages of behavior change.

## Methods

### Research Design and participants

This was a web-based cross-sectional study. Participants were recruited through an automotive company’s health promotion website. The company has branches throughout Japan. The study covered a wide range of professionals involved in occupational health, including managers, clerical workers, and engineers. Data were collected from 979 workers through October 2021. Those who had difficulty making their own decisions due to cognitive impairments were excluded.

All surveys were performed in accordance with the Declaration of Helsinki. Additionally, Based on the “Ethical Guidelines for Life Science and Medical Research for Humans”, the following points were taken into consideration; (1) Appropriately verify the identity of the research participants, (2) Secure opportunities for research participants to ask questions about the content of the explanation and answer them, (3) Participants can read the consent items even after receiving informed consent. All participants were informed about the study in writing before its commencement, and provided informed consent through electromagnetic means. Returning the questionnaire and filling in the checkbox was considered consent for participation. This study was approved by the Institutional Review Board of the Faculty of Medicine, Tokyo Medical and Dental University (approval number M2021-085).

## Measures

Data on demographic characteristics, such as age, gender, occupation, and employment status, were collected at the beginning of the study. In addition to the TSRQ (Diet and Exercise), we assessed the stage of behavior change, which is based on the transtheoretical model (TTM), to measure the scale’s convergent validity.

### The treatment self-regulation questionnaire (TSRQ)

The TSRQ was used to measure participants’ motivation in maintaining diet- and exercise-related behaviors. According to the Center for Self-Determination Theory (CSDT), the original version of the scale consists of 15 items each on Diet and Exercise, and each domain further comprises four subscales (autonomous motivation, introjected regulation, external regulation, and amotivation) [10]. All items are rated on a 7-point Likert scale ranging from 1 (Not at all true) to 7 (Very true). Except for items 5, 10, and 15, the higher the score, the higher the autonomous motivation. Existing research suggests that the validity of the TSRQ and the internal consistency of each subscale is adequate (most α values > 0.73) [9].

### Translation of the TSRQ into Japanese

To translate the scale into Japanese, we first obtained permission from the CSDT to use the TSRQ. A licensed Japanese physician, who was a native Japanese speaker and fluent in English, and who was also well-versed in both Japanese and Western healthcare systems, translated the scale into Japanese. Consistency between the Japanese and English versions of the scale was ensured by (1) using simple sentences, (2) using nouns rather than pronouns, (3) avoiding metaphors and colloquial phrases, (4) avoiding passive expressions, and (5) avoiding hypothetical expressions [22]. In addition, there were discussions between the researchers, who were licensed nurses or public health nurses and physicians, to check whether the wording of an item was appropriate for the field of health guidance and whether participants could understand the item; corrections were made as necessary. Back-translation into English was performed by a Japanese bilingual expert, and the CSDT confirmed the conceptual integrity of the scale’s translated version by reviewing the items.

### The Stages of Behavior Change

The TTM, which is the theory underlying the behavior change stages, was developed in the 1980s [23]. It was introduced to Japan in the late 1990s when the country began to focus on measures to prevent and manage lifestyle-related diseases [24]. Since 2000, studies have applied the TTM to Japanese individuals. The theory is widely used, and the Ministry of Health, Labour and Welfare of Japan also recommends using behavior change stages in health guidance [25]. The stages of change model posits that individuals move through five stages of behavioral change: pre-contemplation, contemplation, preparation, action, and maintenance. Therefore, we asked participants to fit their health behavior to one of the five stages through the following questions: “I have no intention of acting at all” “I plan to act in the future” “Sometimes I act” “Within 6 months since I acted” “Over 6 months since I acted”. The behavior change stage scale used in Japan has been verified—the Cronbach’s alpha coefficients for the Diet items are .74and its reliability and validity have been confirmed [26]. Research on Exercise items has also been reported [27].

### Analysis

We calculated Cronbach’s alpha for internal consistency, Item-Total correlation for examining reliability, conducted correlational analyses for testing convergent validity, and conducted confirmatory factor analysis for structural validity. SPSS version 24 was used for each analysis.

### Internal consistency

According to the COSMIN criteria, the sample size for any analysis of internal consistency is considered “good” if it is five times the number of items and more than 100. Since the Diet and Exercise questionnaires in this study together consist of 30 items, the minimum sample size required was 150. Therefore, the sample size in this study was sufficient and met the COSMIN criteria.

Since previous studies [10] have confirmed that the TSRQ has a four-factor structure (autonomous motivation, introjected regulation, external regulation, and amotivation), the total score on the Japanese version of the TSRQ and the Cronbach’s alpha for each factor were calculated to evaluate internal consistency. In addition, Item-Total correlations (hereinafter referred to as “I-T correlations”) were calculated to examine reliability. In the Japanese version of the TSRQ, items 5, 10, and 15 measure the lack of motivation and were reverse-scored. After performing the I-T correlation, items that were unreliable and unsuitable were excluded.

### Convergent validity

Convergent validity was assessed by calculating Pearson’s correlation coefficients between the TSRQ and the stage of behavior change. The effect size detected in this study was 0.3 [28]. The sample size was calculated using G*Power 3.1. For an alpha error of 0.05 and a power of 0.8, it was estimated that a minimum of 352 participants would be required. Therefore, the sample size for this study was sufficient and met the COSMIN criteria.

### Structural validity

A confirmatory factor analysis (CFA) was performed to assess structural validity. Based on previous studies, a four-factor model was assumed [10]. The COSMIN criterion for the minimum sample size for the factor analysis was met. The maximum likelihood estimation method was used, with the chi-square value (χ^2^), goodness-of-fit of Comparative Fit Index (CFI), and Root Mean Square Error of Approximation (RMSEA). The goodness-of-fit and RMSEA cutoffs were 0.90 or more and 0.08 or less, respectively [29].

## Results

### Participants

Of the 979 participants, 912 (682 males and 230 females) consented to participate and responded to the questionnaire (valid response rate: 93.1%). The demographic characteristics of the participants are shown in Table 1. Participants’ mean age (standard deviation [SD]) was 47.66 (10.51) years. The most common work pattern was day shift (77.63%), and half of the participants (58.60%) were employed in skilled and technical work.

**Table 1:** Participant Demographics

	Total(n = 912)		Male(n = 682)		Female(n = 230)	
	N	%	n	%	n	%
**Age**	M = 47.66 (SD = 10.51)		M = 49.45 (SD = 10.00)		M = 42.37 (SD = 10.23)	
**Work pattern**						
Day shift	708	77.63	489	71.70	219	95.22
Two-shift system	181	19.84	174	25.51	7	3.04
Three-shift system	14	1.64	14	2.05	0	0.00
Other	8	0.88	4	0.59	4	1.74
Unidentified	1	0.11	1	0.15	0	0.00
**Job position**						
Management	166	18.20	163	23.90	3	1.30
Technician	244	26.75	234	34.31	10	4.35
Clerk	210	23.03	112	30.79	98	42.61
Professional engineer	291	31.91	172	25.22	119	51.74
Unidentified	1	0.11	1	0.15	0	0.00

M, mean; SD, standard deviation

## Internal consistency of the Japanese Version of the TSRQ

The mean scores and Cronbach’s alphas of the Japanese version of the TSRQ and its subscales are shown in Table 2. The overall Cronbach’s alpha coefficient for all 15 items in relation to Diet was 0.82, and the Cronbach’s alpha coefficients for its subscales ranged from 0.55 to 0.86. The overall Cronbach’s alpha coefficient for the 15 items in relation to Exercise was 0.81, and the Cronbach’s alpha coefficients for the subscales were 0.58 for amotivation and 0.87 for autonomous motivation.

**Table 2:** Mean Scores and Cronbach’s Alphas for the Japanese Version of the TSRQ

	Item	Mean	SD	Cronbach’s α
TSRQ Diet				
Autonomous motivation	1,3,6,8,11,13	5.61	0.85	0.86
Introjected regulation	2,7	3.77	1.40	0.73
External regulation	4,9,12,14	3.61	1.25	0.82
Amotivation	5,10,15	2.57	1.02	0.55
Amotivation*	5,15	2.10	1.16	0.71
TSRQ Exercise				
Autonomous motivation	1,3,6,8,11,13	5.60	0.90	0.87
Introjected regulation	2,7	3.74	1.47	0.75
External regulation	4,9,12,14	3.31	1.30	0.85
Amotivation	5,10,15	2.35	0.99	0.58
Amotivation*	5,15	1.97	1.08	0.71

TSRQ, treatment self-regulation questionnaire

The I-T correlations are shown in Table 3. For Diet, the I-T correlations ranged from 0.34 to 0.67, except for item 10, whose I-T correlation was low and negative, at –0.15. For Exercise, the I-T correlations ranged from 0.26 to 0.65 except for item 10, whose I-T correlation was low and negative at –0.21.

**Table 3:** Item-Total Correlation (Diet, Exercise)

	Diet	Exercise
1	0.538	0.546
2	0.570	0.559
3	0.532	0.543
4	0.504	0.406
5	0.344	0.38
6	0.666	0.62
7	0.601	0.571
8	0.613	0.645
9	0.343	0.266
10	-0.151	-0.210
11	0.573	0.571
12	0.453	0.486
13	0.409	0.438
14	0.521	0.513
15	0.348	0.256
P < .001		

Subsequently, item 10, which had the lowest I-T correlation and was negative even after considering reversed items, was removed from the set of both Diet and Exercise items after checking the content of the subscales. This is because items with a low I-T correlation are considered idle items that weakly correlate with all items. The Cronbach’s alpha coefficients for the amotivation items, except for item 10, for both Diet and Exercise are indicated using an asterisk in Table 2. The Cronbach’s alphas for the 14 Diet items and the amotivation subscale were 0.85 and 0.71, respectively. The Cronbach’s alphas for the 14 Exercise items and the amotivation subscale were 0.84 and 0.71, respectively.

## Convergent validity of the Japanese Version of the TSRQ

Table 4 shows the correlation coefficients between the scores of the Japanese version of the TSRQ and the stages of behavior change after excluding item 10. The autonomous motivation score for Diet was positively correlated with the stage of behavior change (0.247, p < .001) and negatively correlated with the amotivation score (– 0.258, p < .001). The autonomous motivation score for Exercise was positively correlated with the stage of behavior change (0.195, p < .001) and negatively correlated with amotivation (– 0.197, p < .001).

**Table 4:** Correlations between TSRQ Subscales and Stages of Change: Diet, Exercise

	Autonomous	Introjection	External	Amotivation
Diet	0.247**	0.132**	0.070*	− 0.258**
Exercise	0.195**	0.046	− 0.063	− 0.194**

*p < .05, **p < .001

TSRQ, treatment self-regulation questionnaire.

## Structural validity of the Japanese Version of the TSRQ

**Fig. 1:**
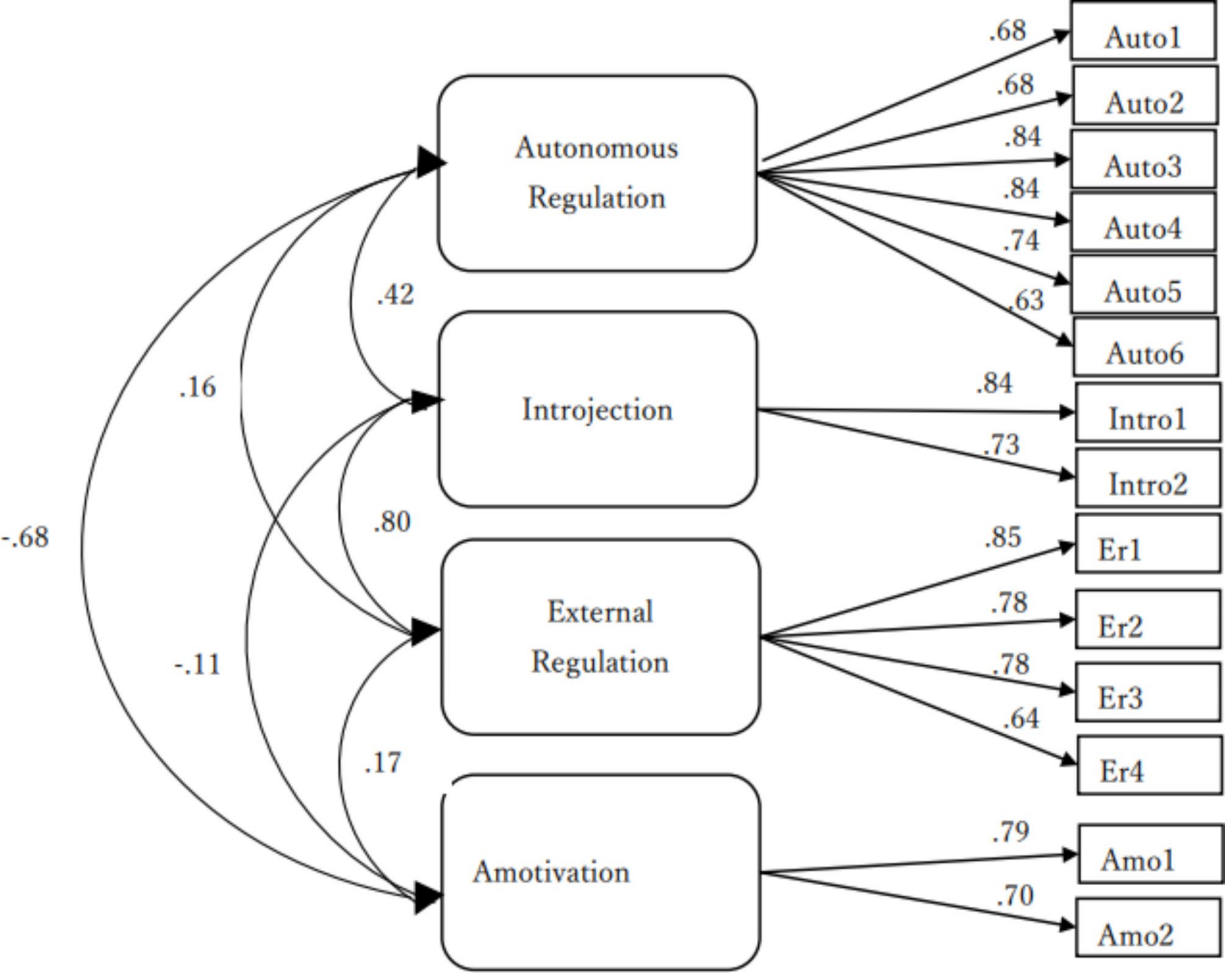
Path Diagram (Exercise)

The CFA results are shown in Table 5, and the Exercise path diagram is shown in Fig. 1. The goodness-of-fit indices of the four-factor hypothesis model were: χ^2^(84) = 574 (p < .001), CFI = 0.93, TLI = 0.89, and RMSEA = 0.08 for Diet and χ^2^(83) = 841 (p < .001), CFI = 0.88, TLI = 0.85, and RMSEA = 0.09 for Exercise.

After removing item 10, which had a low IT correlation, the goodness-of-fit indices of the four-factor hypothesis model were: χ^2^(71) = 392 (p < .001), CFI = 0.94, TLI = 0.92, and RMSEA = 0.07 for Diet, and χ^2^(71) = 558 (p < .001), CFI = 0.92, TLI = 0.91, and RMSEA = 0.08 for Exercise.

**Table 5:** Results of Confirmatory Factor Analysis

	CFI	TLI	RMSEA	χ^²^	df	p-value
Diet	0.94	0.92	0.07	392	71	< 0.001
Exercise	0.92	0.90	0.08	558	71	< 0.001

CFI, Comparative Fit Index; TLI, Tucker Lewis index; RMSEA, Root Mean Square Error of Approximation

## Discussion

### Characteristics of the participants

The purpose of this study was to examine the reliability and validity of the Japanese version of the TSRQ in occupational health settings. In this study, the work patterns included not only daytime work but also shift work. In addition, the study covered a wide range of professionals involved in occupational health including managers, clerical workers, and engineers.

## Internal consistency

For both Diet and Exercise, the overall Cronbach’s alpha coefficient for the 15 items was above 0.70, indicating adequate reliability. Meanwhile, the Cronbach’s alpha coefficients for amotivation, a subscale of both Diet and Exercise, were less than 0.70, indicating low reliability. In addition, item 10—“It is easier to do what I am told by people around me (family, friends, doctors, etc.) than to think about healthy eating by myself”— showed negative I-T correlations for both Diet and Exercise. By removing this item, the Cronbach’s alpha coefficients for amotivation in both Diet and Exercise increased to more than 0.70. The reason for the low reliability of item 10 may be the characteristics of Japanese people [11]. Among Japanese people, self-determination is typically associated with other people and is “situation” dependent [30]. In other words, Japanese people tend to prioritize group values over individual values, depending on the situation. As for item 10, a certain stage of motivation may involve prioritizing, adopting, and implementing behaviors preferred by those around an individual over their own preferences. In other words, it is possible that item 10 does not necessarily indicate a lack of motivation among Japanese people.

## Convergent validity

The convergent validity of the TSRQ was partially confirmed. The scores on the subscales of the Japanese version of the TSRQ were weakly correlated with the stage of behavior change for both Diet (autonomous motivation: r = .247, amotivation: r = − .258) and Exercise (autonomous motivation: r = .195, amotivation: r = − .194). The reason for the low correlation in the present study is that the stage of motivation for behavior change did not necessarily match the actual behaviors or their continuity. As mentioned previously, regardless of the presence or absence of motivation, Japanese people tend to emphasize performing actions that others consider desirable, which we believe is the sole reason for the weak positive correlation between the stage of behavior change and the Japanese version of the TSRQ in the present study.

In addition, in behavior change theory, the main focus is on the factors that predict “future behavior modification” well (e.g., past behavior), and the variables that can control behavior modification are emphasized [31]. Self-efficacy is one of the variables that can influence behavior change [32], and such variables should be explored in future validation studies.

## Structural validity

For Diet, the CFI was 0.93, indicating a good fit. Generally, TLI values range from 0 to 1, and the closer the value is to 1, the better the fit [33]. In this study, the TLI was 0.89, suggesting a good fit. RMSEA, which indicates the discrepancy between the distribution of the model and the true distribution, was 0.08, with the acceptable value being considered as 0.08 or less [34]. Since this value is deemed unfit at 0.10 or more, the value of 0.08 in this study was considered an “almost good fit.” Based on these results, the hypothesized four-factor model for Diet showed a good fit for the 15 items of the original version. For Exercise, the CFI was 0.88, and the TLI was 0.85, suggesting a generally good fit. The RMSEA was 0.09; therefore, it was not a good fit. After removing item 10, which had a weak I-T correlation, both the CFI and the TLI were 0.90 or more, the RMSEA was 0.80 or less, and the degree of fit improved for both Diet and Exercise.

As discussed, by removing item 10, the goodness-of-fit for both Diet and Exercise improved and validity was ensured. Considering Japan’s cultural background, it is necessary to continue paying attention to the interpretation of responses to item 10 in the future.

## Limitations of the study

The first limitation of this study is that the cross-sectional study design precludes retest reliability and measurement error, and therefore, the reliability and validity of this aspect of the study could not be assessed. Second, the study was conducted at a single company, which may have resulted in selection and subject biases. Additionally, we collected almost all questionnaires from companies with branch offices all over the country, but could not obtain information regarding the region the respondents belonged to. Therefore, it was not possible to confirm whether the number of respondents corresponded to the number of people in the area, which affected sampling. Finally, this study only included items for subjective evaluation, and the validity was not assessed through an objective evaluation. In the future, it will be necessary to expand the range of participants and validate the scale using objective indicators, to address these limitations.

## Conclusion

This study investigated the reliability and validity of the Japanese version of the TSRQ, which measures the degree of motivation toward diet- and exercise-related behaviors in occupational health settings. The strengths of this study are twofold. First, this study was the first to verify a scale that can evaluate the effect of “motivational support “, as used in SHG for maintaining and improving health, in Japan. Second, about 900 people from all over Japan participated in the research, which contributes to the generalizability of the TSRQ and confirms a certain degree of reliability and validity. In the future, while considering the removal of items, it is necessary to target a variety of different occupations, to improve the generalizability of the scale and verify its internal consistency. The findings of this study may be used as a basis for promoting primary and secondary prevention. However, empirical studies employing this scale are needed to confirm its value.

## Data Availability

The datasets used and/or analyzed during the current study are available from the corresponding author on reasonable request.

## List of abbreviations

NCDs: Non-communicable diseases
SHC: specific health check-up
SHG: specific health guidance
TSRQ: Treatment Self-regulation questionnaire
SDT: Self-determination theory
